# The Effect of Regional Factors on the Mental Health Status of Frontline Nurses and Patients With COVID-19 During COVID-19: The Role of Depression and Anxiety

**DOI:** 10.3389/fpubh.2022.857472

**Published:** 2022-07-13

**Authors:** Shanguang Zhao, Fangfang Long, Xin Wei, Jianqing Tuo, Hui Wang, Xiaoli Ni, Xin Wang

**Affiliations:** ^1^Centre for Sport and Exercise Sciences, Universiti Malaya, Kuala Lumpur, Malaysia; ^2^Department of Psychology, Nanjing University, Nanjing, China; ^3^Institute of Information Engineering, Xi'an Eurasia University, Xi'an, China; ^4^Institute of Social Psychology, School of Humanities and Social Sciences, Xi'an Jiaotong University, Xi'an, China; ^5^Department of the Psychology of Military Medicine, Air Force Medical University, Xi'an, China; ^6^Strategic Support Force Medical Center, Beijing, China

**Keywords:** nurses, patients, COVID-19, mental wellbeing, depression, anxiety

## Abstract

At the end of 2019, Wuhan, Hubei Province, China, experienced the ravages of Coronavirus disease 2019 (COVID-19). In a few months, infected people rose to tens of thousands. This study aimed to explore the mental health status of military nurse personnel assisting (non-Hubei area) in the fight against COVID-19 and local nurse personnel (in the Wuhan area), as well as the differences in mental health status between nurses and COVID-19 patients that provide a reference basis for psychological crisis intervention. A convenience sampling method was used to select frontline nurses and COVID-19 patients (sample size 1,000+) from two mobile cabin hospitals from January to March 2020. The questionnaire consists of socio-demographic information, Patient Health Questionnaire 9 (PHQ-9), Generalized Anxiety Disorder 7 (GAD-7), General Mental Health Service Questionnaire and Work Intensity and Physical Status Questionnaire. The results showed that depression was present in 117 nurses (19.73%) and 101 patients (23.33%) with PHQ-9 scores >10; anxiety was present in 60 nurses (10.12%) and 54 patients (12.47%) with GAD-7 >10. The anxiety and depression levels of nurses in Wuhan area were higher than those in non-Hubei area. The differences in PHQ-9 and GAD-7 scores were also statistically significant (*p* < 0.001) when comparing patients from different regions, with anxiety and depression rates of 30.19 and 16.04% in local patients and 16.74 and 9.50% in foreign patients. The comparison between nurses and patients showed that the nurses were more depressed than the patients, while the patients were more anxious. Local nurses in Wuhan had a higher workload intensity than aid nurses (77.72 vs. 57.29%). Over 95% of frontline nurses and patients reported that they had not received any form of psychological counseling before the COVID-19 outbreak. 12.87% (26/194) of frontline nurses in Wuhan had a history of taking hypnotic drugs. However, fewer patients (16/212, 7.55%) took medication than frontline nurses. Anxiety and depression levels were far higher among local nurses and patients in Wuhan than in non-Hubei areas. The nurses had higher levels of depression, while the patients had higher anxiety levels. Providing targeted mental health services to healthcare professionals and patients is necessary when experiencing the impact of a major event.

## Introduction

When faced with major emergencies, the population involved in the event often has physical and psychological reactions and may even experience symptoms of post-traumatic stress disorder (1). Firefighters who were on duty during the 9/11 attacks in the United States suffered convulsions, nightmares, and sleep disturbances following their involvement in emergency care (2, 3). According to a World Health Organization (WHO) study, 9% of people who have experienced a crisis event in the past 10 years suffer from a moderate or severe mental disorder, 22% develop depression (4). On January 30th, 2020, the WHO convened an emergency committee on the novel coronavirus epidemic and identified the epidemic in Wuhan, Hubei Province, China, as an international public health emergency (5).

Coronavirus 2019 (COVID-19) is an emerging infectious disease caused by a novel coronavirus with strong infectiveness, high incidence, multiple transmission routes, and widespread epidemiological characteristics (6). There are about 19.8 million confirmed cases of patients with COVID-19 and 5,156,433 cumulative deaths worldwide to date, and few countries have been spared^1^. As the core force in public health emergencies, medical and nursing personnel not only have to face the anxiety and fear of a large number of patients with COVID-19 at the scene of the epidemic but also have to overcome their fear and nervousness of being infected by close contact with patients with COVID-19 (7). In addition, the increased workload and physical strain of wearing physical protective equipment threaten nurses' health (8). The huge workload and psychological pressure could easily lead to different degrees of anxiety, depression, and panic among health care workers. Studies show that 81.8%−92.68% of frontline nurses may have negative emotions due to high work intensity, low experience in responding to public emergencies and lack of protective materials (9). A study also revealed that nursing staff who had cared for suspected or confirmed cases of patients with COVID-19 had significantly higher rates of anxiety and depressive symptoms than the rest of the population (10). Negative emotions can lead to individual stress reactions, which can affect the physical and mental health of health care workers, as well as reduce the quality of work and job satisfaction of health care workers, thus affecting patient outcomes (11, 12).

Due to the severe shortage of medical personnel, medical teams were formed across the country to support Wuhan rapidly. By May 16, 2020, 42,000 medical workers have supported Wuhan, of which nurses account for 68%, far exceeding other medical personnel (13). In the face of the sudden onset and high infectivity of COVID-19, we hypothesize that front-line nurses who assist face greater psychological stress than local front-line nurses. They were more likely to suffer from psychological problems such as guilt, self-blame, insomnia, fear, frustration and powerlessness. In addition, the unfamiliar, high-intensity and high-risk work environment make them also exposed to intense work pressure, which is highly likely to produce negative emotional problems such as depression and anxiety, thus affecting the efficiency of work and causing a certain negative impact on the prevention and control of the epidemic. Chen et al. (14) found that nurses in Taiwan who worked during the outbreak of SARS experienced severe psychological distress. Chen et al. (15), who studied the SARS outbreak, concluded that doctors and nurses exposed to the psychological pressures associated with life-threatening infectious diseases experience high depression and anxiety. Although previous studies have examined the mental health status of frontline nurses, no study has yet described differences in mental health status between nurses in outbreak centers and aid nurses from other provinces.

People diagnosed with COVID-19 are also receiving attention for their mental health and physical pain. In the early stages of the outbreak, individuals with suspected COVID-19 symptoms experienced a variety of mental and psychiatric states (16–18). Wuhan's hospitals were overcrowded before the city's lockdown measures were taken on January 23, 2020. While waiting for diagnosis and treatment, patients are under much psychological stress. Many studies have shown that high psychological and physical stress levels can induce anxiety and depression-like behaviors (19, 20). Previous studies have mainly focused on depression and anxiety levels in patients after infection with infectious diseases (21). According to a study of patients suffering from Middle East Respiratory Syndrome, feelings of anger and anxiety were 16.6 and 7.6%, respectively, in 1,656 patients who were isolated. Part of the anxiety or anger resulted in isolation from family members and friends (22). It is suggested that stress levels of infected patients were raised immediately after infection. Many patients who survived contagious diseases experienced post-traumatic stress disorder (23). However, few studies have examined the emotional differences between patients and nurses. Only a few studies have compared emotional differences between patients and physicians suffering from SARS. Huang et al. (24) included that doctors/nurses infected by SARS experienced fewer emotional disorders than regular patients. Yet, this study's objects were infected medical workers rather than working with the patients, and the sample size is limited.

Therefore, the main purpose of this study was to investigate the influence of regional factors (Wuhan region and non-Hubei region) on the mental health status of nurses and patients, as well as differences in the mental health status of patients and nurses. The results of this study may provide useful information for frontline nurses and patients to develop supportive strategies to improve mental health during the epidemic and timely attention and intervention after the assistance mission.

## Materials and Methods

### Participants

A convenience sampling method was used to conduct the questionnaire survey in two square cabin hospitals (COVID-19) in Wuhan, Hubei Province, China. The survey was conducted from February 10 to March 10, 2020. Front line nurses (including the Wuhan and non-Hubei areas) and patients (including the Wuhan and non-Hubei areas) were invited to participate in the survey. All questionnaires were distributed and collected on-site at the hospital by doctors.

The Ethics Committee of the Air Force Medical University approved this study (CBA20200315). All procedures were performed in accordance with the ethical standards set by the Declaration of Helsinki.

### Measures

#### Demographic Form

A self-developed questionnaire was used to investigate the demographic information of nurses, including gender, age, educational level, professional title, clinical experience, working duration as a frontline nurse, average working hours per shift, whether Wuhan is the original working place, whether the current department is intensive care unit (ICU). The demographic information of patients consisted of age, education background, occupation, and time of admission to the temporary shelter hospital.

### Mental Health Assessment

The Patient Health Questionnaire 9 (PHQ-9) and Generalized Anxiety Disorder 7 (GAD-7) are the quantitative assessment criteria for mental health recommended by the Diagnostic Statistical Manual of Mental Disorders, fifth edition (DSM-V), published by the American Psychiatric Association and have good reliability and validity (25). The depression and anxiety status of participants were evaluated using PHQ-9 and GAD-7, a quick and easy-to-administer screening tool for depression and anxiety, respectively, that is widely used in clinical settings (26, 27).

The PHQ-9-Chinese version was used to assess depression, with nine items self-report instrument, divided into four grades, almost no = 0, some days = 1, more than half = 3, almost every day = 4. Each question is scored from 0 to 3 according to the frequency in the preceding 2 weeks, and a higher score reflects poorer conditions. The total score of PHQ-9 ranged from 0 to 27, in which 0 to 5 was not depressed, 6 to 9 were mild, 10 to 14 was moderate, 15 to 19 was severe, and 20 to 27 was very severe. The PHQ-9 uses a score of 10 as the cutoff value indicating depression (28), and Cronbach's α coefficient is between 0.8 and 0.9 (29).

The GAD-7-Chinese version is a brief self-rating scale of anxiety symptoms developed by Spitzer et al. (30) based on DSM-V to assess the frequency of anxiety symptoms in the past 2 weeks. The GAD-7 scale consists of 7 items on a 4-point scale, not at all = 0, a few days = 1, more than half of the days = 2, and almost every day = 3 (31). The reliability and validity of this scale can effectively reflect the anxiety and degree of the subjects (26).

### General Mental Health Service Questionnaire

In order to avoid the participant's anxiety and depression survey results from being affected by the mental health wellbeing that may exist previously before being infected of COVID-19 or before the outbreak, and the possible existing psychological counseling relationship, this study also investigated the possible mental health service happening of the subjects. The survey contains seven questions. Have you received professional psychological assistance before? If so, is the consultation paid or free? What kind of practitioner is your consultant? Is the consultation face-to-face or online? Is the consultation accepted in Hubei or other provinces? And are you taking sleep aids or antidepressants or anxiety drugs?

#### Nurse's Work Intensity and Physical Status

Taking into account the work situation and physical health of the first-line nurses and the wellbeing of their family members have greatly affected their mental health. A set of questionnaires for these questions was also distributed to the nurses' group. This set of questionnaires investigated the subjects' physical health in detail, including whether or not there were symptoms of suspected infection in the past week. And the intensity of the nurse's work in the past week, including specific working hours and night shifts.

### Procedure

For each subject who participated in the survey, their basic information was first collected using a demographic form. The PHQ-9 and GAD-7 were then completed. To avoid the influence of psychological counseling that may exist before the outbreak of COVID-19, the study also used unstructured questionnaires to investigate participants' mental health services before the outbreak. In addition, we also used unstructured questionnaires to investigate the working situation, physical health status and happiness of family members of front-line nurses, which may have an impact on their mental health. The two non-structured questionnaires were assessed and tested by six experts in the field, and the items with content validity index (CVI) >0.78 and Kappa value >0.74 were retained. The final questions are listed in Items in **Tables 5**, **6**.

### Statistical Analysis

The Kolmogorov–Smirnov test was used to examine the normal distribution of continuous variables. The mean ± Standard error (Mean ± SE) was used for data with a normal distribution, whereas the median was used for data with non-normal distribution. Differences in demographic and clinical characteristics were tested using the Chi-square tests or two samples independent sample *t*-tests. All the data was performed and analyzed by SPSS 20.0 (IBM Corporation, New York, USA).

## Results

### Demographic Characteristics

A total of 552 valid questionnaires were collected in the analyses with a recovery rate of 92%, including 195 questionnaires from local nurses in Wuhan and 357 assisted nurses from non-Hubei regions. Of the 552 frontline nurses, 513 (93.11%) were female, of which 188 (96.53%) were from Wuhan, and 325 (91.3%) were from non-Hubei areas. The majority (89.31%) of nurses were aged between 20 and 40 years, and the number of young nurses from non-Hubei areas was approximately twice as many as in Wuhan. All of them have college-level or education or higher. Current work hours show that the number of nurses working in their current work unit for more than 8 weeks is the highest (*n* = 118, 21.38%). More than half of the nurses in Wuhan (58.91%) and non- Hubei regions (59.34%) have <10 years of experience. Eighty-nine frontline nurses reported that they were transferred from their former workplaces after the COVID-19 outbreak. The demographic information of the nurse is shown in Table 1.

**Table 1:** Demographics characteristics of nurses on admission.

**Basic information**	**Wuhan *N*/(%)**	**Non-Hubei *N*/(%)**
Gender (Female)	195 (96.53%)	357 (91.3)
**Age**		
20~30	98 (48.51)	197 (50.38)
30~40	72 (35.64)	126 (32.23)
40~50	25 (12.38)	46 (11.76)
>50	7 (3.47)	22 (5.63)
**Education**		
College	78 (38.61)	116 (29.67)
Undergraduate	119 (58.91)	264 (67.52)
Master	5 (2.48)	11 (2.81)
**Time participates the current work (weeks)**		
1	4 (1.98)	52 (13.3)
2	34 (16.83)	31 (7.93)
3	20 (9.9)	24 (6.14)
4	23 (11.39)	85 (21.74)
5	23 (11.39)	66 (16.88)
6	23 (11.39)	37 (9.46)
7	21 (10.4)	10 (2.56)
8	9 (4.46)	13 (3.32)
>8	45 (22.28)	73 (18.67)
**Professional qualifications**		
Nurse practitioner	64 (31.68)	120 (30.69)
Nurse	72 (35.64)	110 (28.13)
Supervisor's career	54 (26.73)	132 (33.76)
Deputy director's nurse	10 (4.95)	25 (6.39)
Chief nurse	2 (0.99)	4 (1.02)
**Years of work**		
**≤10 years**	119 (58.91)	232 (59.34)
11~20	50 (24.75)	87 (22.25)
21~30	22 (10.89)	50 (12.79)
**>30 years**	11 (5.45)	22 (5.63)
**Previous department**		
Internal medicine	88 (43.56)	32 (8.18)
Men's section	0 (0)	1 (0.26)
Psychiatry	1 (0.5)	122 (31.2)
Emergency department	19 (9.41)	20 (5.12)
ICU	5 (2.48)	5 (1.28)
General branch	10 (4.95)	4 (1.02)
Imaging section	1 (0.5)	0 (0)
Laboratory section	0 (0)	1 (0.26)
Surgical department	37 (18.32)	15 (3.84)
Rehabilitation department	6 (2.97)	153 (39.13)
Logistics department	5 (2.48)	6 (1.53)
Gynecologic	6 (2.97)	3 (0.77)
Pediatric	9 (4.46)	9 (2.3)
Oncology	3 (1.49)	5 (1.28)
Infectious department	6 (2.97)	3 (0.77)
Chinese medicine	2 (0.99)	10 (2.56)
Five official sections	3 (1.49)	1 (0.26)
Dermatology	1 (0.5)	1 (0.26)
**Level the hospital**		
3A	158 (78.22)	267 (68.29)
3B	3 (1.49)	67 (17.14)
2A	15 (7.43)	51 (13.04)
2B	26 (12.87)	6 (1.53)
**Current department**		
ICU	9 (4.46)	7 (1.79)
Non-ICU	193 (95.54)	384 (98.21)
**Change the working place**		
No	156 (77.23)	348 (89)
Yes	46 (22.77)	43 (11)

A total of 450 questionnaires were distributed to patients, of which 433 (96.2%) valid questionnaires were returned, including 212 (48.96%) patients from Wuhan and 221 (51.04%) patients from non-Hubei regions (Table 2). More female patients than male patients from Wuhan (52.80%) and non-Hubei regions (55.70%). The age of these patients ranged from 18 to 60 years. The demographic information of the patients is shown in Table 2.

**Table 2:** General information of interviewees on admission.

**Basic information**	**Wuhan (*n* = 212)**	**Non-Hubei (*n* = 221)**	***p*** **-Value**
Gender (Female)	52.80%	55.70%	0.555
**Age**			
18–35	33.00%	50.70%	0.001
36–60	61.80%	45.20%	
>60	5.20%	4.10%	
**Education**			
Senior high school and below	21.70%	10.40%	<0.0001
Secondary	26.90%	10.40%	
College	23.10%	24.40%	
Undergraduate	24.50%	39.40%	
Master and above	3.80%	15.40%	
**Occupation**			
Family of medical staff	0.50%	14.50%	<0.0001
Retirement	17.50%	5.00%	
Student	2.40%	3.60%	
Individual businesses	11.80%	9.00%	
Employee	30.20%	21.70%	
Farmer	1.90%	2.30%	
Other	35.60%	43.90%	
Arrival time at the temporary shelter hospital (weeks, mean, SD)	2.36, 0.62	—	

### PHQ-9

The statistical results of the PHQ-9 questionnaire are shown in Table 3. The results of the PHQ-9 for the nurse population showed a significant difference between nurses from Wuhan and the non-Hubei region (*p* < 0.001). Both the total score and the scores of each question were significantly different. This result indicates that Wuhan nurses are more depressed than non-Hubei nurses. Moreover, the number of local nurses in Wuhan with PHQ-9 scores higher than 10, the threshold for depression, was higher than the number of nurses outside Hubei province.

**Table 3:** Outcomes of PHQ-9 for nurses and patients (Mean ± SE).

**Items**	**Nurses**		***p*** **-Value**	**Patients**		***p*** **-Value**
	**Wuhan (*n* = 202)**	**Non-Hubei (*n* = 391)**		**Wuhan (*n* = 212)**	**Non-Hubei (*n* = 221)**	
1. Little interest or pleasure in doing things	1.01 ± 0.06	0.64 ± 0.04	<0.0001	0.73 ± 0.04	0.76 ± 0.06	0.679
2. Feeling down, depressed, or hopeless	0.91 ± 0.06	0.48 ± 0.03	<0.0001	0.92 ± 0.05	0.56 ± 0.05	<0.0001
3. Trouble falling or staying asleep, or sleeping too much	1.35 ± 0.07	0.76 ± 0.04	<0.0001	1.24 ± 0.07	0.83 ± 0.06	<0.0001
4. Feeling tired or having little energy	1.23 ± 0.07	0.71 ± 0.04	<0.0001	1.17 ± 0.05	0.83 ± 0.06	<0.0001
5. Poor appetite or overeating	1.1 ± 0.06	0.66 ± 0.04	<0.0001	0.95 ± 0.05	0.65 ± 0.06	0.0001
6. Feeling bad about yourself or that you are a failure or have let yourself or your family down	0.75 ± 0.06	0.43 ± 0.04	<0.0001	0.92 ± 0.06	0.52 ± 0.05	<0.0001
7. Trouble concentrating on things, such as reading the newspaper or watching television	0.88 ± 0.06	0.46 ± 0.04	<0.0001	0.92 ± 0.06	0.65 ± 0.04	0.0007
8. Moving or speaking so slowly that others could have noticed, or being so fidgety/restless that you have been moving more than usual	0.73 ± 0.06	0.36 ± 0.03	<0.0001	0.87 ± 0.05	0.48 ± 0.05	<0.0001
9. Thoughts you would be better off dead, or of hurting yourself	0.49 ± 0.06	0.22 ± 0.03	<0.0001	0.60 ± 0.05	0.33 ± 0.04	<0.0001
Total score	8.44 ± 0.47	4.71 ± 0.26	<0.0001	8.31 ± 0.28	5.60 ± 0.39	<0.0001
	***N*** **(%)**	***N*** **(%)**		***N*** **(%)**	***N*** **(%)**	
**Total PHQ ≥10**	63 (31.19%)	54 (13.81%)	<0.0001	64 (30.19%)	37 (16.74%)	0.0009
Total PHQ 0–4	59 (29.21%)	229 (58.57%)	<0.001	33 (15.57%)	113 (51.13%)	<0.001
Total PHQ 5–9	80 (39.6%)	108 (27.62%)		115 (54.25%)	71 (32.13%)	
Total PHQ 10–14	3 3 (16.34%)	29 (7.42%)		43 (20.28%)	19 (8.60%)	
Total PHQ 15–19	15 (7.43%)	20 (5.12%)		19 (8.96%)	12 (5.43%)	
Total PHQ 20–27	15 (7.43%)	5 (1.28%)		2 (0.94%)	6 (2.71%)	

*Clinically, the answers to these questions are assigned a score between 0 and 3 (from 0 for “not at all” to 3 for “nearly every day”)*.

*For a total range of 0–27. CUT-OFF 10*.

The results of the PHQ-9 for the patient population showed significant differences in the total PHQ-9 scores between patients from Wuhan and non-Hubei regions (*p* < 0.001). For single items, the differences were significant for 2–8 questions except for the first question (*p* = 0.679), indicating that local patients in Wuhan were significantly more depressed than those in non-Hubei areas. The number of local patients with total PHQ-9 scores higher than 10 was higher than those in non-Hubei regions.

**Figure 1:**
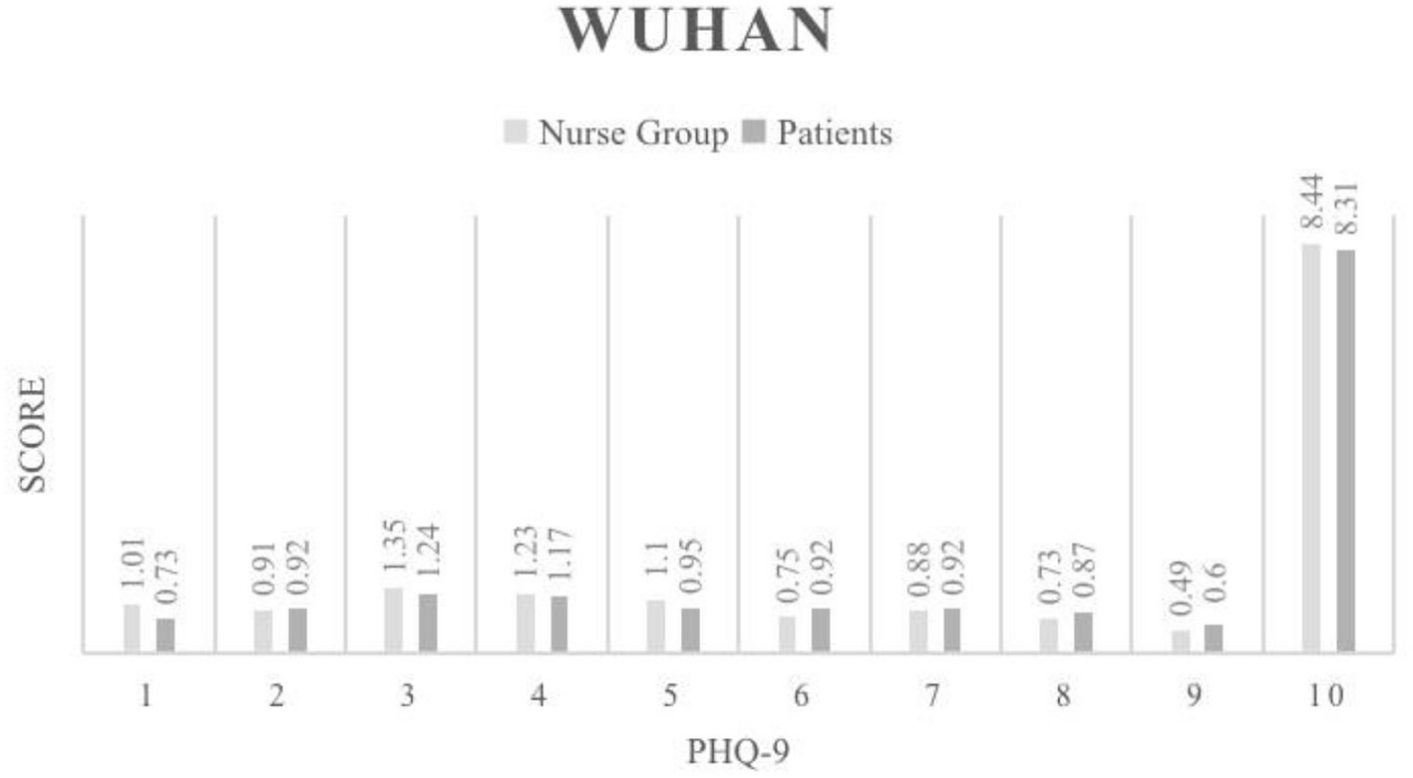
PHQ-9 Phq-9 score results for the Wuhan region.

**Figure 2:**
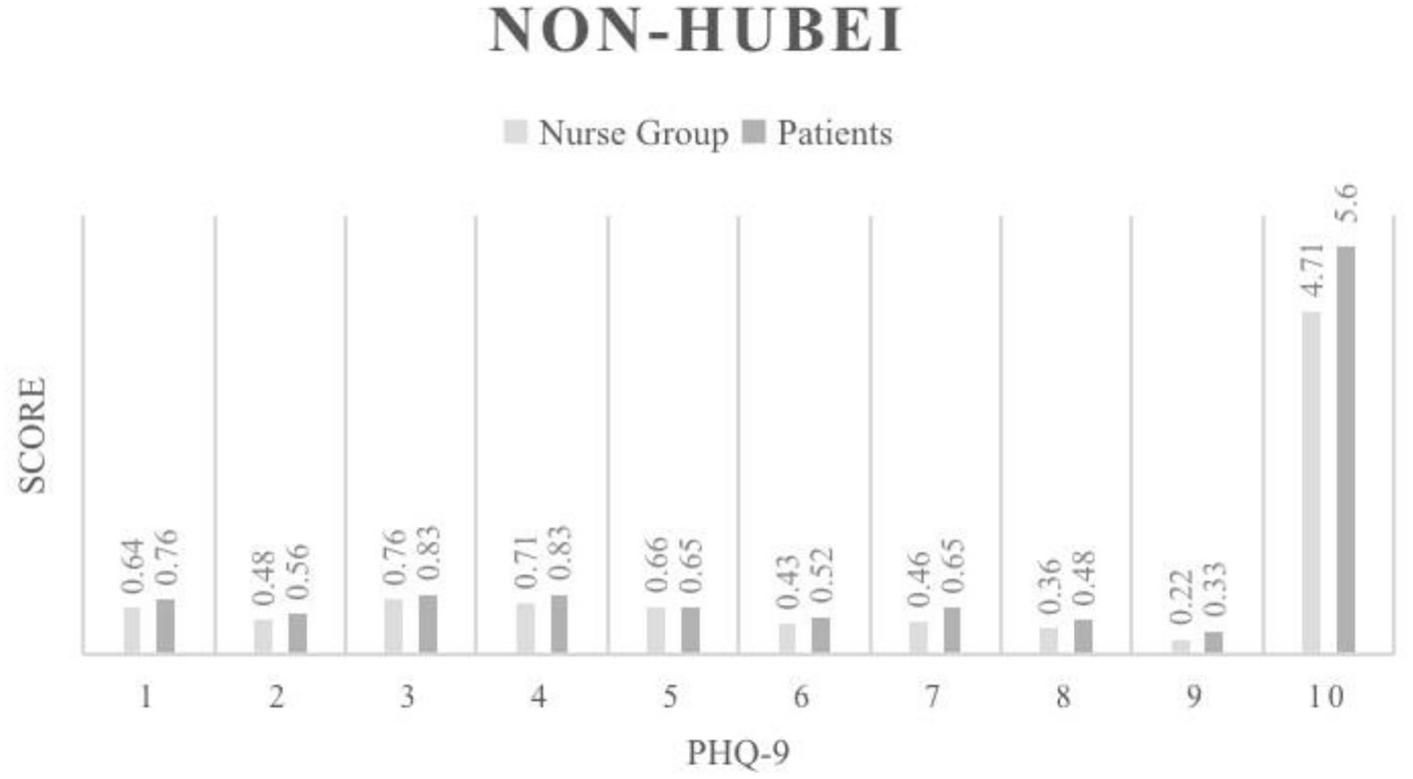
PHQ-9 score results for the Non-Hubei region.

Our study also compared PHQ-9 score between nurses and patients in the Wuhan area (Figure 1). The results showed that the average depression score of nurses was 8.44, while that of patients was 8.31, showing no statistically significant difference (*p* > 0.05). In the non-Hubei region (Figure 2), the average depression score of nurses was 4.71, and that of patients was 5.6, and there was no statistically significant difference between them (*p* > 0.05).

### GAD-7

The results of the GAD-7 for the nurse population showed a significant difference between nurses from the Wuhan area and nurses from non-Hubei areas (*p* < 0.001). Significant differences were demonstrated in both the total score and the score of each question. Moreover, the anxiety level of local nurses in Wuhan was higher than that of nurses in non-Hubei areas. The results of the GAD-7 for the patient population showed significant differences between patients in Wuhan and non-Hubei regions (*p* < 0.001). Significant differences were demonstrated in both the total score and the score of each question. Anxiety was significantly greater in local Wuhan patients than in non-Hubei regions.

**Figure 3:**
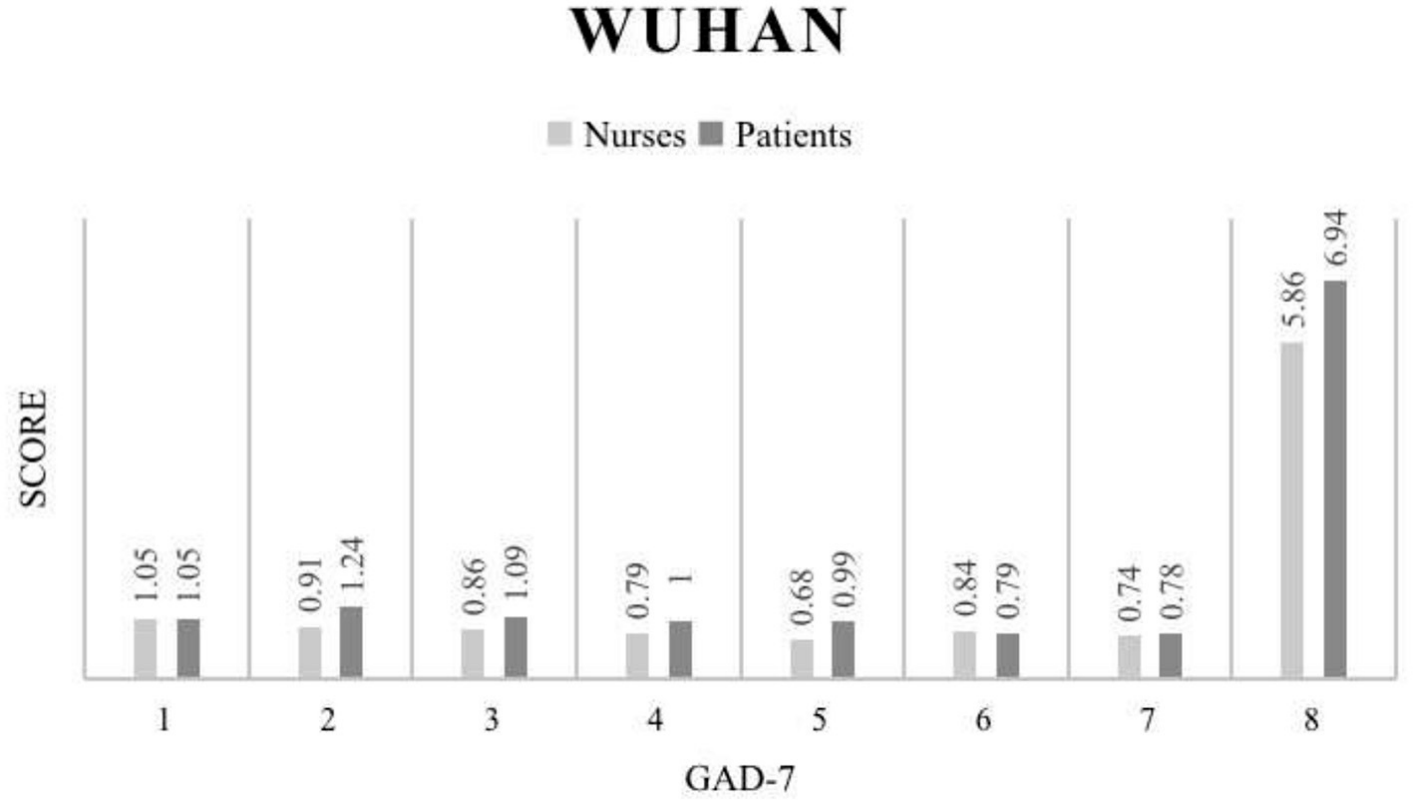
GAD-9 score results for the Wuhan region.

**Figure 4:**
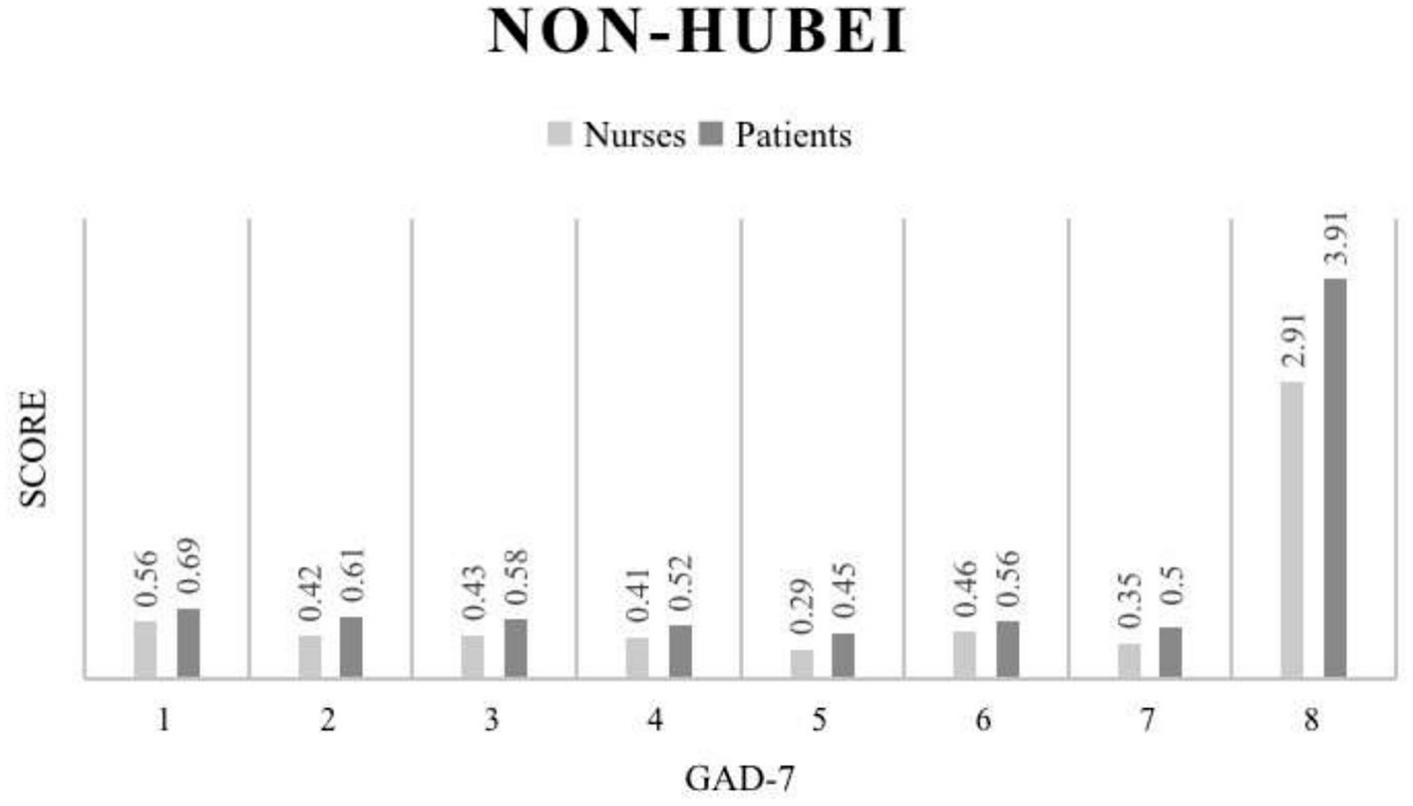
GAD-7 score results for the Non-Hubei region.

Our study also compared the GAD-7 of nurses and patients in Wuhan (Figure 3), and the results showed that the average anxiety score of nurses and patients was 5.86 and 6.94, respectively, indicating that the anxiety of local patients was higher than that of local nurses. In non-Hubei region (Figure 4), the average anxiety score of nurses was 2.91, and that of patients was 3.91. The anxiety degree of non-Hubei patients was higher than that of non-Hubei nurses (Table 4).

**Table 4:** Outcomes of GAD-7 for nurses and patients (Mean ± SE).

**Items**	**Nurses**		***p*** **-Value**	**Patients**		***p*** **-Value**
	**Wuhan (*n* = 202)**	**Non-Hubei (*n* = 391)**		**Wuhan (*n* = 212)**	**Non-Hubei (*n* = 221)**	
1. Feeling nervous, anxious, or on edge	1.05 ± 0.06	0.56 ± 0.03	<0.0001	1.05 ± 0.05	0.69 ± 0.05	<0.0001
2. Not being able to stop or control worrying	0.91 ± 0.06	0.42 ± 0.03	<0.0001	1.24 ± 0.06	0.61 ± 0.05	<0.0001
3. Worrying too much about different things	0.86 ± 0.06	0.43 ± 0.03	<0.0001	1.09 ± 0.06	0.58 ± 0.05	<0.0001
4. Trouble relaxing	0.79 ± 0.06	0.41 ± 0.03	<0.0001	1.00 ± 0.06	0.52 ± 0.05	<0.0001
5. Being so restless that it's hard to sit still	0.68 ± 0.06	0.29 ± 0.03	<0.0001	0.99 ± 0.06	0.45 ± 0.05	0.002
6. Becoming easily annoyed or irritable	0.84 ± 0.06	0.46 ± 0.03	<0.0001	0.79 ± 0.05	0.56 ± 0.05	0.0001
7. Feeling afraid as if something awful might happen	0.74 ± 0.06	0.35 ± 0.03	<0.0001	0.78 ± 0.06	0.50 ± 0.05	<0.0001
Total score	5.86 ± 0.40	2.91 ± 0.20	<0.0001	6.94 ± 0.22	3.91 ± 0.30	<0.0001
**Total score ≥10**	37 (18.32%)	23 (5.88%)		34 (16.04%)	20 (9.50%)	0.041
Total score 0–4	91 (45.05%)	277 (70.84%)		45 (21.23%)	134 (60.63%)	<0.0001
Total score 5–9	74 (36.63%)	91 (23.27%)		133 (62.74%)	65 (29.86%)	
Total score 10–13	9 (4.46%)	8 (2.05%)		24 (11.32%)	7 (3.17%)	
Total score 14–18	17 (8.42%)	12 (3.07%)		90 (4.25%)	11 (4.98%)	
Total score 19–21	11 (5.45%)	3 (0.77%)		10 (0.47%)	3 (1.36%)	

*CUT-OFF 10*.

### Working Intensity and Physical Fitness Outcomes of the Frontline Nurses

The results showed that most nurses did not show fever and other symptoms of COVID-19 infection in the past 2 weeks, but they felt strong physical discomfort, including sore throat and dyspepsia. In terms of work intensity, both local nurses in Wuhan and nurses who assisted Hubei in the field experienced higher intensity work than before the outbreak. It is worth emphasizing that the local nurses in Wuhan felt a higher workload intensity than the nurses who assisted Hubei (77.72 vs. 57.29%). In terms of self-assessment, the obvious fatigue is statistically significant in Wuhan local nurses and the nurses who assisted Hubei. Table 5 shows the frontline nurses' working intensity and physical fitness outcomes.

**Table 5:** Work intensity and physical condition of nurses.

**Items**	**Wuhan**	**Non-Hubei**	***p*** **-Value**
	**(%)/*N***	**(%)/*N***	
**Do you have a fever in the last 2 weeks?**			
No	195 (96.53)	388 (99.23)	0.0363
Yes	7 (3.47)	3 (0.77)	
**Do you have respiratory symptoms in the last**			
**2 weeks?**			
No	164 (81.19)	386 (98.72)	<0.0001
Yes	38 (18.81)	5 (1.28)	
**Do you have systemic symptoms in the last 2 weeks?**			
No	179 (88.61)	387 (98.98)	<0.0001
Yes	23 (11.39)	4 (1.02)	
(A) (None)			
No	67 (33.17)	24 (6.14)	<0.0001
Yes	135 (66.83)	367 (93.86)	
(B) Sore throat			
No	154 (76.24)	375 (95.91)	<0.0001
Yes	48 (23.76)	16 (4.09)	
(C) Anti-acid reflux			
No	196 (97.03)	389 (99.49)	0.0213
Yes	6 (2.97)	2 (0.51)	
(D) Indigestion			
No	185 (91.58)	387 (98.98)	<0.0001
Yes	17 (8.42)	4 (1.02)	
(E) Diarrhea			
No	189 (93.56)	387 (98.98)	0.0004
Yes	13 (6.44)	4 (1.02)	
(F) Constipation			
No	182 (90.1)	382 (97.7)	0.0002
Yes	20 (9.9)	9 (2.3)	
(G) Bloated			
No	192 (95.05)	389 (99.49)	0.0006
Yes	10 (4.95)	2 (0.51)	
(H) Abdominal pain			
No	198 (98.02)	389 (99.49)	0.1875
Yes	4 (1.98)	2 (0.51)	
Other			
No	192 (95.05)	385 (98.47)	0.015
Yes	10 (4.95)	6 (1.53)	
**In the last 2 weeks, did your lung CT show**			
**any signs of “ground glass shadow”?**			
No	196 (97.03)	391 (100)	0.0015
Yes	6 (2.97)	0 (0)	
**The last month, there has been change work position?**			
No	44 (21.78)	151 (38.62)	<0.0001
Yes	158 (78.22)	240 (61.38)	
**On average, how many day shifts are in a week?**			
0–2	70 (34.65)	145 (37.08)	0.7223
3–5	99 (49.01)	178 (45.52)	
>5	33 (16.34)	68 (17.39)	
**On average, how many night shifts are in a week?**			
0–2	129 (63.86)	329 (84.14)	<0.0001
3–5	68 (33.66)	52 (13.3)	
>5	5 (2.48)	10 (2.56)	
What is the average length of work per shift?			
<8 h	142 (70.3)	157 (40.15)	<0.0001
8–16 h	59 (29.21)	228 (58.31)	
17–24 h	1 (0.5)	6 (1.53)	
**Compared to the outbreak before, about the last 2**			
**weeks of your work intensity, you think:**			
It's not very different	45 (22.28)	167 (42.71)	<0.0001
It's harder than before	92 (45.54)	169 (43.22)	
Significantly harder than before	65 (32.18)	55 (14.07)	
**Have you taken the following isolation measures?**			
No	39 (19.31)	271 (69.31)	<0.0001
Self-isolation at home	22 (10.89)	68 (17.39)	
Separation from the family	141 (69.8)	52 (13.3)	
**Are there any family members in your home**			
**who need to be cared for?**			
No	80 (39.6)	196 (50.13)	0.0002
Older person	42 (20.79)	40 (10.23)	
Infants or children	66 (32.67)	143 (36.57)	
Pregnant women	0 (0)	2 (0.51)	
People with disabilities	1 (0.5)	0 (0)	
Other needs to be taken care of	13 (6.44)	10 (2.56)	
**In the last 2 weeks, have your family**			
**had respiratory symptoms?**			
No	187 (92.57)	385 (98.47)	0.0002
Yes	15 (7.43)	6 (1.53)	
No	200 (99.01)	391 (100)	0.1157
Yes	2 (0.99)	0 (0)	
**According to your feelings and experience**,			
**how often does the following situation appear to you?**			
Work makes me feel physically and mentally exhausted			
Never	12 (5.94)	95 (24.3)	<0.0001
Occasionally	89 (44.06)	226 (57.8)	
Regularly	52 (25.74)	49 (12.53)	
Frequently	16 (7.92)	13 (3.32)	
Daily	33 (16.34)	8 (2.05)	
I feel exhausted after work			
Never	12 (5.94)	89 (22.76)	<0.0001
Occasionally	82 (40.59)	209 (53.45)	
Regularly	54 (26.73)	68 (17.39)	
Frequently	19 (9.41)	15 (3.84)	
Daily	35 (17.33)	10 (2.56)	
I feel very tired when I wake up in the morning			
and have to face a day of work			
Never	30 (14.85)	165 (42.2)	<0.0001
Occasionally	78 (38.61)	179 (45.78)	
Regularly	52 (25.74)	28 (7.16)	
Frequently	11 (5.45)	12 (3.07)	
Daily	31 (15.35)	7 (1.79)	
I doubt the significance of the work I do			
Never	93 (46.04)	284 (72.63)	<0.0001
Occasionally	71 (35.15)	82 (20.97)	
Regularly	19 (9.41)	17 (4.35)	
Frequently	5 (2.48)	4 (1.02)	
Daily	14 (6.93)	4 (1.02)	
**According to your feelings and experience, how**			
**often does the following situation appear to you?**			
**I can effectively solve problems at work**			
Never	2 (0.99)	13 (3.32)	0.0424
Occasionally	19 (9.41)	27 (6.91)	
Regularly	94 (46.53)	144 (36.83)	
Frequently	25 (12.38)	54 (13.81)	
Daily	62 (30.69)	153 (39.13)	
I feel I am making contribution to the hospital			
Never	3 (1.49)	10 (2.56)	0.0252
Occasionally	28 (13.86)	27 (6.91)	
Regularly	73 (36.14)	129 (32.99)	
Frequently	20 (9.9)	34 (8.7)	
Daily	78 (38.61)	191 (48.85)	
In my opinion, I am good at my job			
Never	2 (0.99)	13 (3.32)	0.0034
Occasionally	25 (12.38)	21 (5.37)	
Regularly	79 (39.11)	137 (35.04)	
Frequently	28 (13.86)	47 (12.02)	
Daily	68 (33.66)	173 (44.25)	
I am confident that I can do all the work effectively			
Never	2 (0.99)	10 (2.56)	0.0088
Occasionally	17 (8.42)	15 (3.84)	
Regularly	79 (39.11)	127 (32.48)	
Frequently	29 (14.36)	46 (11.76)	
Daily	75 (37.13)	193 (49.36)	

### Previous Mental Health Condition

In this study, more than 95% of frontline nurses reported that they had not received any form of psychological counseling before the COVID-19 outbreak. But it is worth noting that 12.87% of the frontline nurse (26/194) have a history of taking hypnotic drugs in Wuhan. More than 95% of patients reported that they had not received any form of mental health counseling in the past. However, fewer patients were taking medication compared with the frontline nurses (16/212). From an objective point of view, this sudden outburst has become the main cause of their psychological problems. The results of the mental health history are shown in Table 6.

**Table 6:** Mental health service for nurses and patients.

**Items**	**Nurses (Mean ±SE)**		**Patients**	
	**Wuhan (*N* = 202)**	**Non-Hubei (*N* = 391)**	**Wuhan (*N* = 212)**	**Non-Hubei (*N* = 221)**
**Have you received professional psychological assistance? (%):**				
Yes	194 (96.04 %)	375 (95.91%)	11 (5.2%)	9 (4.1%)
No	8 (3.96%)	16 (4.09%)	201 (94.8%)	212 (95.9%)
**What kind of professional psychological assistance have you received? (%):**				
No	194 (96.04 %)	375 (95.91%)	**/**	**/**
Paid service	1 (0.5%)	10 (2.56%)		
Free service	7 (3.47%)	11 (02.81%)		
**What kind of expert's assistance have you received? (%):**				
No	194 (96.04%)	378 (96.68%)	**/**	**/**
Psychiatrist	0	2 (0.51%)		
Counselor	6 (2.97%)	8 (2.05%)		
Social worker	1 (0.5%)	2 (0.51%)		
Other	1 (0.5%)	1 (0.26%)		
**What kind of psychological assistance has been received? (%)**				
No	194 (96.04%)	378 (96.68%)	**/**	**/**
On-site consultation	3 (1.49%)	6 (1.53%)		
Online consultation	5 (2.48%)	7 (1.79%)		
**What sources of psychological assistance have you received? (%)**				
No	194 (96.04%)	80 (96.19%)	**/**	**/**
Doctors in Hubei Province	3 (1.49%)	1 (0.26%)		
Doctors outside Hubei Province	5 (2.48%)	10 (2.56%)		
**Do you take a sedative or hypnotic drugs? (%):**				
Yes	176 (87.13%)	374 (95.65%)	15 (7.08%)	8 (3.62%)
No	26 (12.87%)	17 (4.35%)	197 (92.92%)	213 (96.38%)
**Do you take antidepressant and anxiety drugs? (%):**				
Yes	195 (96.53%)	387 (98.98%)	8 (3.77%)	4 (1.81%)
No	7 (3.47%)	4 (1.02%)	204 (96.23%)	217 (98.19%)

*/ Too few people to count*.

## Discussion

In this study, we investigated the characteristics of the influence of geographical factors on the mental health status of newly crowned patients and nurses; secondly, we compared the differences in the mental health status of nurses and patients. The results revealed that local nurses and patients in Wuhan had much higher levels of anxiety and depression than in non-Hubei areas; nurses and patients showed different characteristics, with reports indicating higher levels of depression among nurses and higher levels of anxiety among patients.

Previous studies of the SARS and Ebola epidemics have shown that sudden, immediately life-threatening illnesses result in significant stress for health care workers (32). Front-line nurses, who require close contact with patients, confront serious problems such as heavy workload, shortage of protective equipment, fear of infection from family and physical exhaustion, which have a major influence on their physical and mental health (33). A meta-analysis that included 13 cross-sectional studies with a total of 33,062 participants found that during the COVID-19 pandemic, a large proportion of h frontline nurses experienced severe levels of anxiety, depression, and insomnia (34). The prevalence of affective symptoms was higher for women and nurses than for men and physicians. The nurse population is mainly female, so the incidence of affective symptoms is higher than that of physicians. During a COVID-19 outbreak, nurses are often at greater risk of exposure.

### Survey of Regional Factors on Nurses' and Patients' Mental Health

This study is the first comparative study on the psychological status of nurses supporting Hubei province and local nurses. According to the results of PHQ-9, Wuhan local nurses show a significantly higher degree of depression than all non-Hubei nurses in all nine items. The average score of the total has even nearly doubled in the local nurses, most likely because of the increasing number of patients, the shortage of medical resources and the shortage of medical staff in Wuhan before the arrival of foreign aid teams. The long and intensive work made the local nurse experience unprecedented pressure.

Similarly, compared with the record of zero infection in the foreign medical aid teams (34, 35), there was an infection in Wuhan local nurses who lacked protection early stage of the disease. The same similar conclusion is also reflected in the anxiety level of the frontline nurses. In all seven questions of GAD-7, the nurses in the Wuhan are more anxious than the nurses from outside Hubei province, and the total score of GAD-7 is also nearly doubled. These data indicate that anxiety and depression often coexist with health caregivers in high-intensity and high-risk work, coherent with previous studies (36–39).

According to the history of psychological counseling and medication use reported that nurses from other provinces have more experience with psychological counseling than nurses in Wuhan. Nevertheless, the vast majority of nurses have no experience with psychological counseling. The report also showed that in terms of drug use, more nurses in the Wuhan were more likely to choose sleep aids and antidepressants or anti-anxiety medications, suggesting that when faced with more intense stress, the Wuhan nurses preferred assistance with drugs rather than psychological counseling services. It is possible that nurses lacked time and availability for psychological counseling during the epidemic; therefore, this study recommends introducing online counseling services to field nurses.

The same number of male and female respondents were reported based on patient demographic information, with a wide age range. In general, however, relatively more middle-aged and older infected individuals participated in this study, which is consistent with this COVID-19 infection epidemiological survey (40–42). There were no significant differences in occupation or educational background in the infected population. The transmission characteristics of foreign outbreaks also reported no clear trend of virus infection for specific occupations and educational backgrounds (43, 44).

In terms of depression, Wuhan patients showed more obvious depression mood than eight non-Hubei patients in eight of the nine questions in the PHQ-9 survey, and the total score statistics also significantly surpassed the latter. This is related to the time of onset of Hubei patients and the time in line to wait for the hospital admission. In addition, it should also be noted that these local Wuhan patients lacked awareness of the COVID-19 at the early stage of the epidemic outbreak. When they developed symptoms, they received only normal fever or other treatments, and when the disease worsened, a series of physical and psychological changes occurred. The results of anxiety and depression show matching. But it is worth to be mentioned that the anxiety level of Wuhan patients is slightly lower than that of patients in other regions of China and slightly higher than the data of depression level of both. A reasonable explanation is that when a patient receives treatment, more emotions about their condition and the outside world are reflected in the level of anxiety rather than depression. To confirm this conclusion, more research needs to be done. According to the history of psychological counseling and drug use reported, there is no obvious difference between Wuhan patients and patients in other places of China. This also objectively shows that the COVID-19 virus does not tend to these aspects. And the mental health of patients in Wuhan is not different from patients in other parts of China.

### Comparison of Differences in Mental Health Status Between Nurses and Patients

We first compared the results of frontline nurses and patients in Wuhan (Figure 1). The results of the PHQ-9 scores showed that frontline nurses in Wuhan had slightly higher levels of depression than patients. In particular, there was a statistically significant difference between the frontline nurses and patients in terms of loss of interest in other things, which objectively indicates a higher level of depression among both patients and health care workers in Wuhan, but the overall situation was worse for overworked nurses. From a psychological support perspective, both populations need counseling and encouragement, and these results provide a reference for countries with high epidemic prevalence, such as EU member states and the United States, which are experiencing a severe test of COVID-19. According to the statistical results of the non-Hubei area, both nurses and patients in the non-Hubei area had significantly lower PHQ-9 scores than the Wuhan (Figure 2). However, patients in the non-Hubei were more likely to be anxious than nurses. It is reasonable to explain that nurses in the non-North Lake group had better protection and peer nurses supported each other. But when a patient is infected, fear of the unknown disease develops. However, patients in the non-Lakeland group were more likely to be anxious than nurses, and a reasonable explanation is that nurses in the non-Lakeland group had better protection and peer nurses supported each other. When a patient is infected, there is fear of the unknown disease. Figure 3 below shows the specific data.

Amusingly, the results for anxiety appear to be the opposite of the results for depression. According to the data of the Wuhan area, the patients have more obvious anxiety than the nurses. This emotion is particularly reflected in the inability to control anxiety, inability to sleep, and irritability. There was a statistically significant difference between patients and nurses on the total score. A reasonable guess at this set of figures is that nurses work hard and don't have much time to think about other things, whereas patients are unable to contact their family members in the hospital, but seeing more and more patients enter the hospital makes them more restless.

The results of the GAD-7 data comparison between non-Hubei patients and nurses reveal that more statistically significant differences can be observed (Figure 3), further confirming the reasonable speculation of anxiety mentioned above. Although the overall level of anxiety was not as pronounced as in Wuhan, the anxiety status of patients in non-Hubei areas was still a cause for concern. These results suggest that with limited resources for psychological support, priority is given to psychological support for people in the hardest-hit areas. For example, in the European Union region (45), Italy's Lombardy region (46), and New York City in the United States (47), this COVID-19 infection high-risk patients require anxiety relief work. Figure 4 below shows the specific data of the non-Hubei class.

Sudden public events cause varying mood swings in those affected by them, and some symptoms of depression and anxiety disorders emerge. At the end of 2019, an outbreak of novel coronavirus pneumonia in Wuhan caused a huge shock on residents, healthcare professionals, families and patients in different areas. According to the findings of this survey, the intense workload and risk of infection following the outbreak caused both local and field nurses to experience significant depression and anxiety, with local nurses in Wuhan showing more pronounced depression and anxiety due to their long working hours and lack of protection in the early days. Although health care professionals did not face significant risks and other problems, patients admitted to the hospital also had significant anxiety and depression. According to the survey results, after the outbreak, local nurses in Wuhan showed more pronounced depression and anxiety than nurses in non-Hubei areas due to their long early working hours and lack of protection. Although medical workers and other issues face no major risks, the admitted COVID-19 patients also felt significant anxiety and depression. By comparing the nurses and patients in the high-risk and low-risk areas, it was clear that medical staff and patients in the high-risk area experienced more intense emotional distress. That can explain why some nurses in Italy chose to commit suicide after learning they were infected with the virus under stressful work conditions and lack of medical supplies (48). In addition, even in areas where the epidemic was not prominent, psychological changes occurred among medical staff and patients, with patients exhibiting significant anxiety. These data support the development of future psychological work in public emergencies.

### Limitation and Future Outlook

This study adopted a survey method and the sample size exceeded 1,000. However, Wuhan's COVID-19 patients and full-time nurses far exceed this number from a research perspective. Therefore, the sample size covers a small range. In addition, this study mainly focuses on the influence of regional factors on the mental health status of nurses and patients, as well as the differences between patients and nurses. However, in real life, not only regional factors may affect the anxiety and depression of these subjects. Although we have collected demographic variables such as age, education level and working years, we have not conducted further analysis on these variables in this paper. Future studies can comprehensively consider the impact of regional factors and other demographic factors on people's psychological status in the context of the epidemic.

In terms of clinical intervention, although research surveys show that both the nurse group and the patient group are suffering from depression and anxiety, the counseling interventions that can be done are very limited. One reason is that the nurses were very busy during the epidemic and did not have time to receive professional psychological counseling. Another reason is that the flow of nurses and patients is obvious, and it is difficult to track the follow-up status of subjects after a one-time questionnaire. Although various provinces in China have sent a certain number of psychological counseling workers to support Wuhan, the number of patients who can receive counseling is limited, and this number is even less for medical staff. The SARS that broke out in China in 2003 has put the country through a test. The lack of related psychological counseling services has prompted the investigation of psychological counseling services in this COVID-19 epidemic. Although it is not yet possible for everyone to receive psychological support, it is believed that more and more psychological support will be given to people fighting the disease on the road of human anti-epidemic. Up to now, more and more countries in the world have been violently impacted by the COVID-19, and China's successful anti-epidemic experience is worth promoting and learning. Hopefully, other countries will lead the way in terms of psychological support.

## Data Availability Statement

The raw data supporting the conclusions of this article will be made available by the authors, without undue reservation.

## Ethics Statement

The Ethics Committee of the Air Force Medical University approved this study (CBA20200315). The patients/participants provided their written informed consent to participate in this study.

## Author Contributions

Conceptualization: HW. Methodology: FL, XN, and SZ. Software: JT. Writing—original draft preparation: SZ. Writing—review and editing: XWei, XWang, FL, and SZ. Funding acquisition: XWang. All authors contributed to the article and approved the submitted version.

## Conflict of Interest

The authors declare that the research was conducted in the absence of any commercial or financial relationships that could be construed as a potential conflict of interest.

## Publisher's Note

All claims expressed in this article are solely those of the authors and do not necessarily represent those of their affiliated organizations, or those of the publisher, the editors and the reviewers. Any product that may be evaluated in this article, or claim that may be made by its manufacturer, is not guaranteed or endorsed by the publisher.

## Footnotes

^1^ https://coronavirus.jhu.edu/
